# Role of amino acid infusion in delayed recovery from neuromuscular blockers

**DOI:** 10.4103/0019-5049.63649

**Published:** 2010

**Authors:** Seema Kalra, Rachna Wadhwa

**Affiliations:** Department of Anaesthesiology and Critical Care, IGESI Hospital, Jhilmil, Delhi-110 095, India

**Keywords:** Amino acid, hypothermia, residual neuromuscular blockade

## Abstract

This case report highlights the anaesthetic management of a patient who had residual muscle paralysis following neuromuscular blockade, which was attributed to hypothermia and corrected by administration of amino acid solution. The various causes of residual neuromuscular blockade should be considered when treating such a patient. Amino acid infusion has been found to hasten the recovery from neuromuscular block due to vecuronium bromide aggravated by hypothermia.

## INTRODUCTION

Delayed awakening from anaesthesia could be due to many causes, e.g. overdose of intravenous and volatile anaesthetics, neuromuscular blocking drugs, opioids, hypoxia, hypothermia, electrolyte and endocrinal abnormalities. Hypothermia is a common cause of delayed recovery from anaesthesia. Mild hypothermia also decreases drug metabolism and has also been shown to prolong postoperative recovery.[1] Our case report suggests that that the infusion of amino acid solution hastened the recovery from residual neuromuscular blockade.[2,3] Previous studies have reported that intravenous amino acid infusions exert enhanced thermogenic effects during general anaesthesia.[2]

## CASE REPORT

A 65-year-old, 45-kg woman diagnosed with cholelithiasis presented for cholecystectomy. She was taken up for surgery with ASA Grade 1, with all preoperative investigations including routine electrocardiogram (ECG) and X-ray of the chest being within normal limits. She was given balanced general anaesthesia. Premedication given consisted of oral ranitidine 50 mg and alprazolam 0.25 mg given on the night before the surgery and 2 hours prior to the surgical procedure. The patient received fentanyl 2 μg/kg, 5 minutes before induction. She was induced with propofol 2 mg/kg, and vecuronium bromide 0.1 mg/kg was given to facilitate endotracheal intubation and the airway was secured with a cuffed oral endotracheal tube of size 7.5. Anaesthesia was maintained with nitrous oxide 66% in oxygen and 0.8% isoflurane. Ventilation was controlled to maintain normocapnia. Continuous monitoring of Electrocardiography, Saturation of Oxygen, Endtidal carbon dioxide and Noninvasive blood pressure was done. The surgery lasted for 1 hour. Total dose of vecuronium given was 4.8 mg. The patient was haemodynamically stable during the intraoperative period. At the end of the surgery, the residual neuromuscular blockade was reversed with atropine 0.02 mg/kg and neostigmine 0.06 mg/kg. The patient was administered Inj. aceclofenac 150 mg IM 20 minutes before closure for postoperative analgesia and ondansetron 4 mg IV to prevent postoperative nausea and vomiting. The patient was sedated but responding to verbal commands, had good respiratory efforts with adequate tidal volume and was maintaining her vitals in the normal range. The trachea of the patient was extubated and the patient was observed in postoperative recovery. The patient continued to have stable vital signs. After 45 minutes, the patient was found to be deeply sedated not responding to painful stimuli, had a shallow respiration and a respiratory rate of 8 breaths per minute, pulse of 68/min, blood pressure of 118/76 mmHg, SpO_2_ was 100%. The patient was shifted to the operation theatre, given 100% oxygen and was connected to the vital signs monitor. The pulse of the patient was 60 beats per minute with few ventricular ectopics, blood pressure was 116/76 mmHg, SpO_2_ was 100% but muscle tone was not good. Thinking in terms of recurarisation and residual weakness from neuromuscular block, neostigmine 1.2 mg was repeated with atropine, lignocaine was given at a dose of 2 mg/kg. Ectopics were corrected. 10 ml of 10% calcium gluconate was given slowly IV, but there was no change in the condition of the patient. Simultaneously the following investigations were carried out: haemogram, blood sugar, blood urea, serum creatinine, liver functions, serum electrolytes and arterial blood gas analysis.

However, the patient continued to be deeply sedated, tidal volume was inadequate, extremities were cold (nasopharyngeal core temperature was measured and found to be 35°C), but she was maintaining arterial oxygen saturation, and did not need reintubation at any time. Forced air warming with a Bair hugger of the patient was started and she was given warm intravenous fluids to correct hypothermia. Arterial blood gas analysis of the patient revealed respiratory acidosis. Amino acid infusion was given at 100ml per hour. The patient remained haemodynamically stable and gradually, after one and a half hour the respiration became regular, respiratory rate was 14 breaths per minute and tidal volume became adequate. She started responding to verbal commands and had a good skeletal muscle tone. Postoperative period was uneventful and the patient was subsequently discharged on the fifth postoperative day.

## DISCUSSION

Causes of delayed awakening could be the residual effects of anaesthetic drugs which could be due to a number of reasons. Excess drug dosage may have been given or the patient is unduly susceptible. Frail, small or elderly patients generally require lower doses than fit, normally sized adults. Delayed drug metabolism occurs in renal or hepatic failure, and smaller doses may be required. Hypoventilation is a frequent cause of delayed emergence. Residual neuromuscular blockade is another important cause which results in paralysis which may be perceived as unresponsiveness though the patient may be fully conscious and aware.

Patients who do not breathe effectively during or after anaesthesia may become hypercarbic (raised CO_2_) to a level that may produce sedation or even unconsciousness[4] Metabolic disturbances like hypoglycemia, hyperglycemia, electrolyte imbalance may also cause delayed recovery from muscle relaxants. Severe hypothermia may lead to reduced consciousness level. A core temperature of less than 33°C has a marked anaesthetic effect itself and will potentiate the central nervous system (CNS) depressant effects of anaesthetic drugs.

The core temperature typically decreases by about 0.5–1.0°C shortly after the induction of anaesthesia.[5] Core hypothermia impairs hepatic and renal metabolic function, so drugs like neuromuscular blocking agents may accumulate.[2] In addition, hypothermia prolongs the duration of action of muscle relaxants as it decreases nerve conduction time, delays repolarisation of nerve spike potential, slows the rate of acetylcholine release, decreases receptor affinity and reduces the rate of muscle Contraction.[6] Overdose of anaesthetic drugs, hypoxia, electrolyte imbalance and hypoglycemia were ruled out.

Several drugs that quicken recovery from neuromuscular blockade caused by vecuronium in anesthetized patients are Ulinastatin and Gabexate mesilate protease inhibitors and Milrinone, a phosphodiesterase III inhibitor Ulinastatin, is thought to promote the release of acetylcholine at the neuromuscular junction and increase hepatic blood flow and urine volume. For this reason, ulinastatin quickens recovery from neuromuscular blockade in anesthetized patients receiving vecuronium. During a continuous infusion of gabexate mesilate, recovery from neuromuscular blockade is quickened. Milrinone, a phosphodiesterase III inhibitor, is supposed to increase the release of acetylcholine at the neuromuscular junction and make the neuromuscular junction sensitive to acetylcholine. Therefore, recovery from neuromuscular blockade is hastened. Nicorandil enhances membrane K+ conductance in skeletal muscle and increases contraction of the skeletal muscle and quickens recovery from neuromuscular blockade.[3]

The patient was a frail, elderly lady who probably had recurarisation (as patient was alright for 45 minutes) associated with opioid effects and mild hypothermia. The patient had a slow respiratory rate of 8 breaths per min which implies opioid induced respiratory depression, but she had received fentanyl almost two hours back so the respiratory depression was unlikely due to opioids. However for inadequate neuromuscular recovery the patient should have had tachypnoea, and desaturation which she did not have, she only had shallow respiration. The Arterial oxygen saturation was always 100%. Monitoring of residual neuromuscular paralysis by a peripheral nerve stimulator would have helped in detection of residual paralysis. So, it becomes mandatory to routinely monitor neuromuscular blockade in every patient, especially when there is a clinical suspicion of delayed recovery and because there are lack of reliable clinical signs of adequate recovery from neuromuscular blockade. Mild degree of hypothermia further worsened the situation compounding the delayed recovery from neuromuscular blocking drugs and maybe decreasing the drug metabolism.[2]

In earlier studies, amino acid infusions have been used successfully to reverse the delayed recovery from neuromuscular blockers, especially when it is associated with any degree of hypothermia,[7] and we did find that after administration of amino acids (150 ml over one and a half hour), the patient became alert, started responding to verbal commands with good skeletal muscle tone. Gupta N *et al*.[8] in their study found that infusion of amino acids @ 100ml/ hour speeds the recovery from vecuronium induced neuromuscular blockade in anaesthetized patients. This could be probably explained by the fact that amino-acid–enriched solutions supply energy to the skeletal muscles, causing an increase in muscle strength and hastening recovery from neuromuscular blockade by generating heat in the skeletal muscles and thus improving the hypothermia during general anaesthesia,[3]

Another way to prevent the development of hypothermia is to stimulate endogenous heat production by administration of amino acid infusion[9]. Amino acids infused during general anaesthesia exert a thermic effect that is fivefold enhanced compared with in the awake state, and may prevent postoperative hypothermia[9]. Previous studies have found that IV amino acid infusions exert enhanced thermogenic effects during general anaesthesia[5]. The mechanism behind this phenomenon is not fully understood, although nutrient intake stimulates energy expenditure, and hence heat production, in the awake state. Although the mechanism is still incompletely understood, a central metabolic inhibitory pathway may be involved.[10,11] During general anaesthesia, the hypothalamic thermoregulation is depressed, and this inhibitory pathway is silenced. Hence, the thermal response to amino acid administration is exaggerated.

Laproscopic cholycystectomy in this patient would have been a better choice as it would preserve the respiratory mechanics. This case report brings out the implications of hypothermia on residual neuromuscular block following vecuronium bromide and the possible role of an infusion of amino acid solution in hastening its recovery.
